# Supplementary figures and images for: Host-defence caerin 1.1 and 1.9 peptides suppress glioblastoma U87 and U118 cell proliferation through the modulation of mitochondrial respiration and induce the downregulation of CHI3L1
Source: PLoS One. 2024 Jun 7;19(6):e0304149. doi: 10.1371/journal.pone.0304149 (PMC11161062; doi:10.1371/journal.pone.0304149)

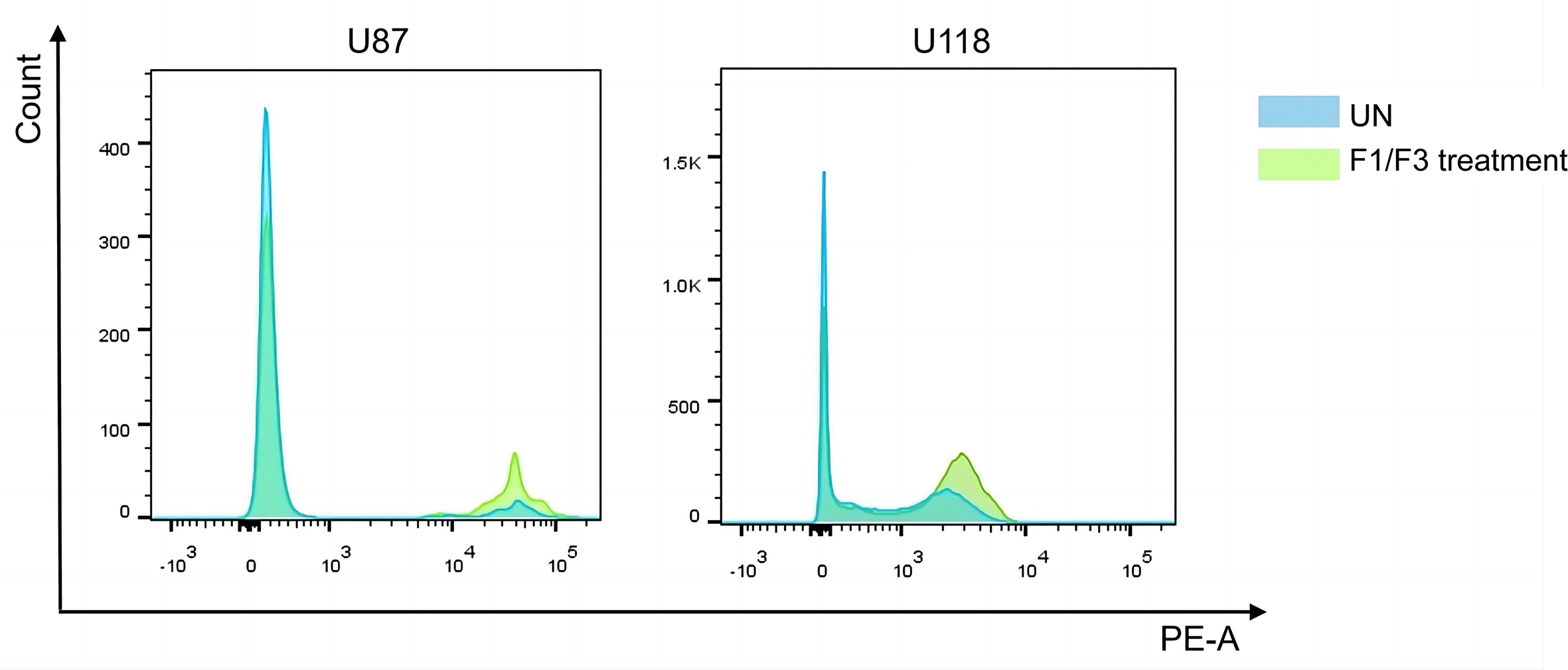

Supplement: S1 Fig — The concentration of F1/F3 was 5 and 10 μg/ml in the treatment of U87 and U118 cells, respectively. (TIF) [file pone.0304149.s001.tif]

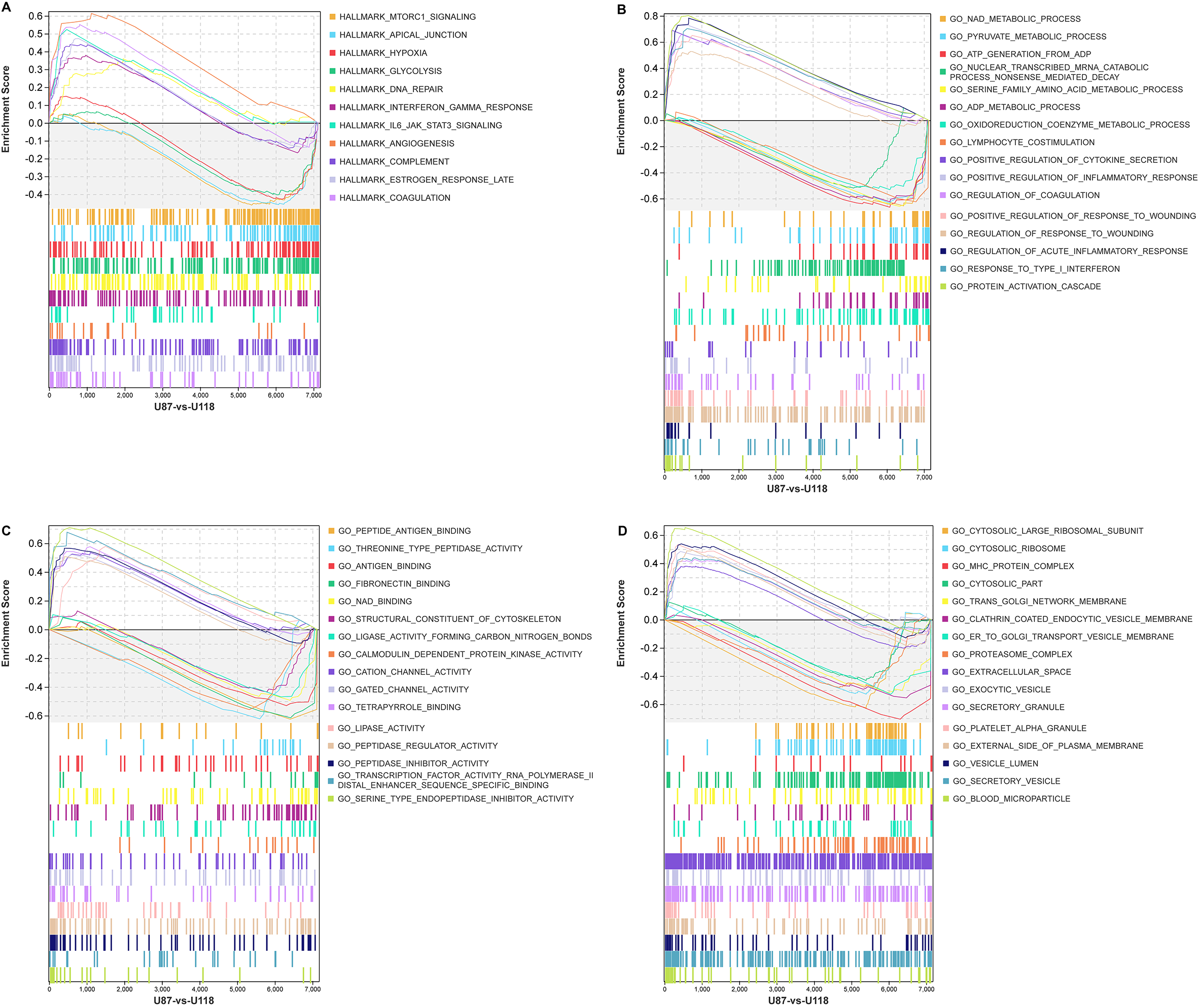

Supplement: S2 Fig — (A) GSEA of the Hallmark pathways in the U87 relative to the U118 groups. Gene ontology terms enriched in between the U87F and U118 groups: (B) biological process; (C) molecular function; and (D) cellular component. (TIF) [file pone.0304149.s002.tif]

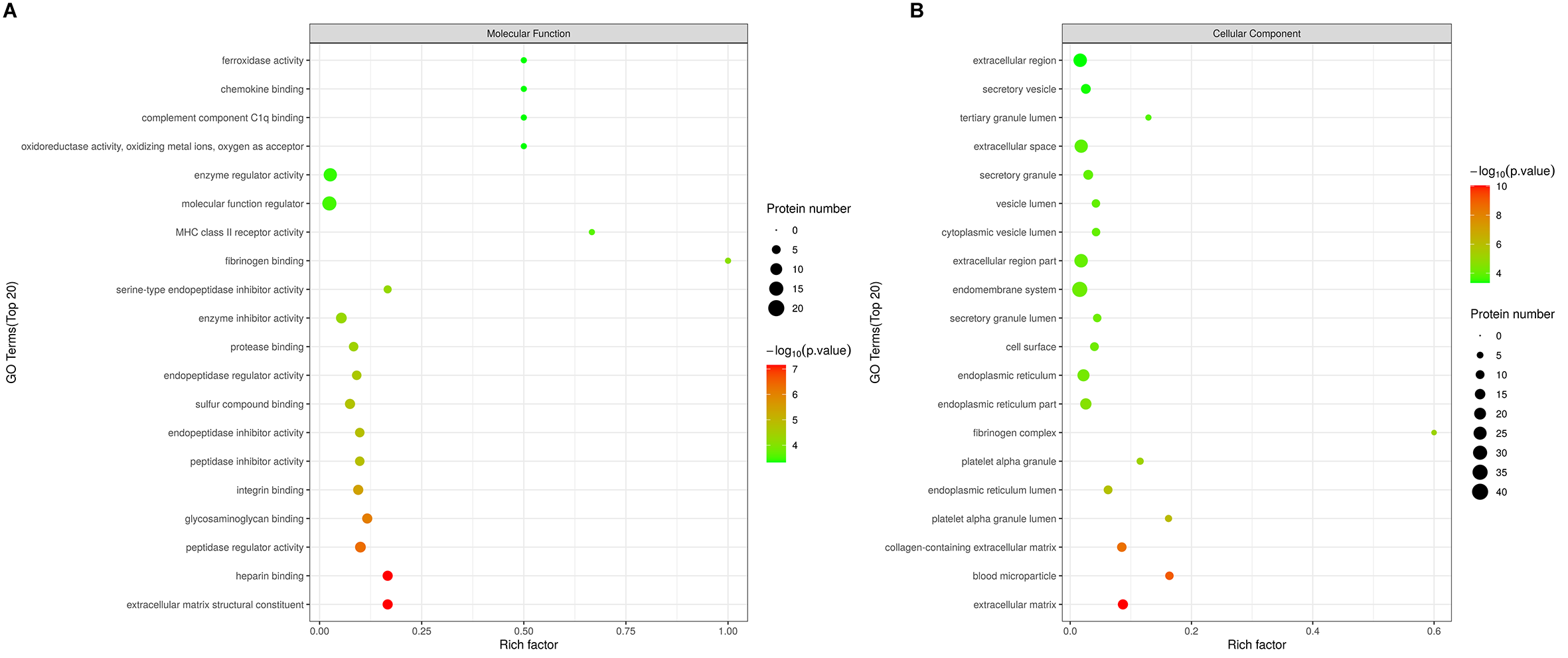

Supplement: S3 Fig — Top 20 molecular function terms (A) and cellular component terms (B) enriched in the U87F group with respect to the U87 group. (TIF) [file pone.0304149.s003.tif]

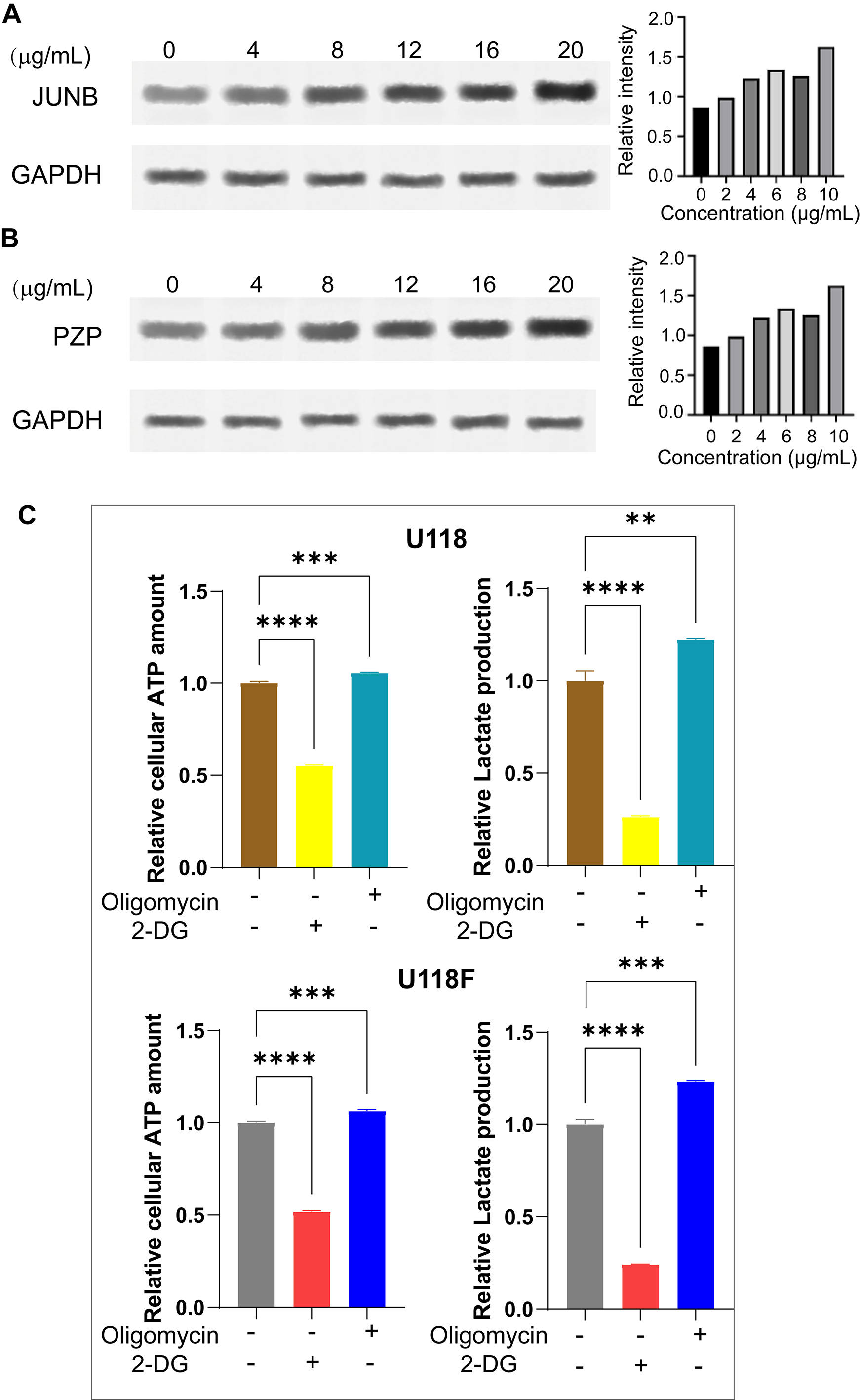

Supplement: S4 Fig — (A) and PZP (B) regulation in the U87 cells treated with different concentrations of F1/F3. (C) Evaluation of glycolysis/OXPHOS levels in the U118 (top) and U118F (bottom) groups. **: P-value < 0.01, ***: P-value < 0.001, and ****: P-value < 0.0001. (TIF) [file pone.0304149.s004.tif]
