# Supplementary material for: Host-defence caerin 1.1 and 1.9 peptides suppress glioblastoma U87 and U118 cell proliferation through the modulation of mitochondrial respiration and induce the downregulation of CHI3L1
Source: PLoS One. 2024 Jun 7;19(6):e0304149. doi: 10.1371/journal.pone.0304149 (PMC11161062; doi:10.1371/journal.pone.0304149)
Supplement: S1 Raw images — (PDF) [file pone.0304149.s005.pdf]

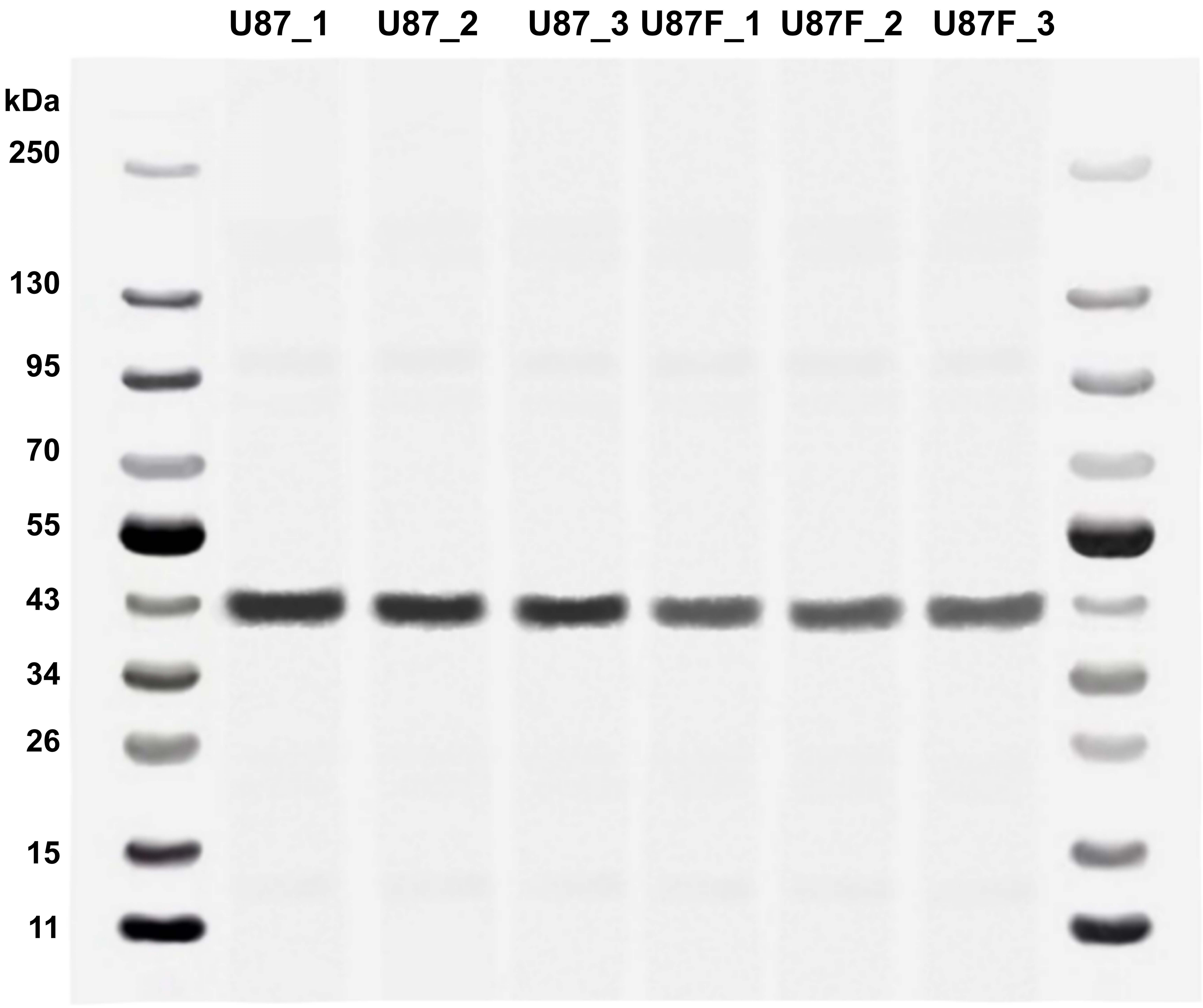

Fig 7A CHI3L1

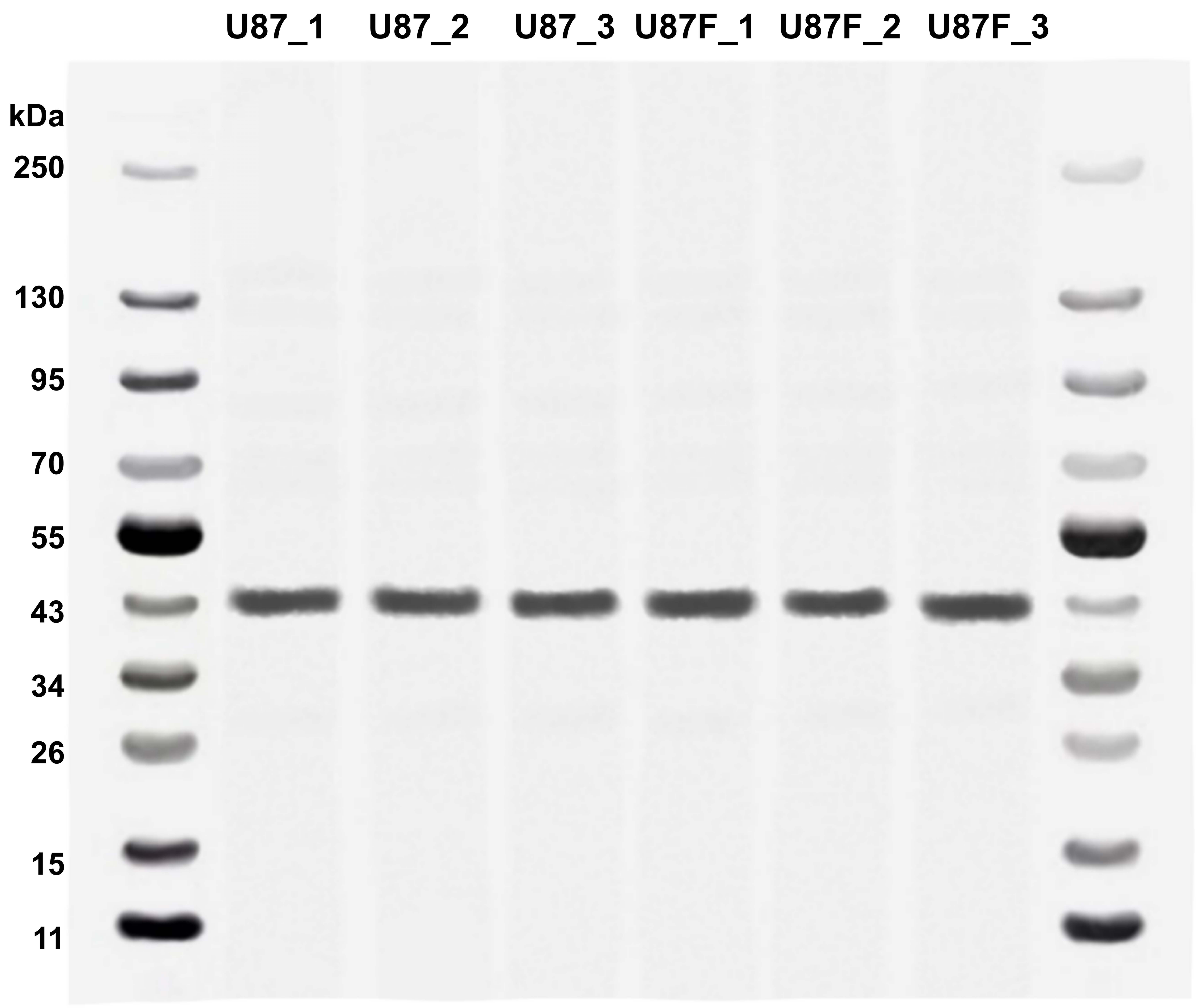
U87_1
U87_2
U87_3
U87F_1
U87F_2
U87F_3
kDa
250
130
95
70
55
43
34
26
15
11

Fig 7A GAPDH

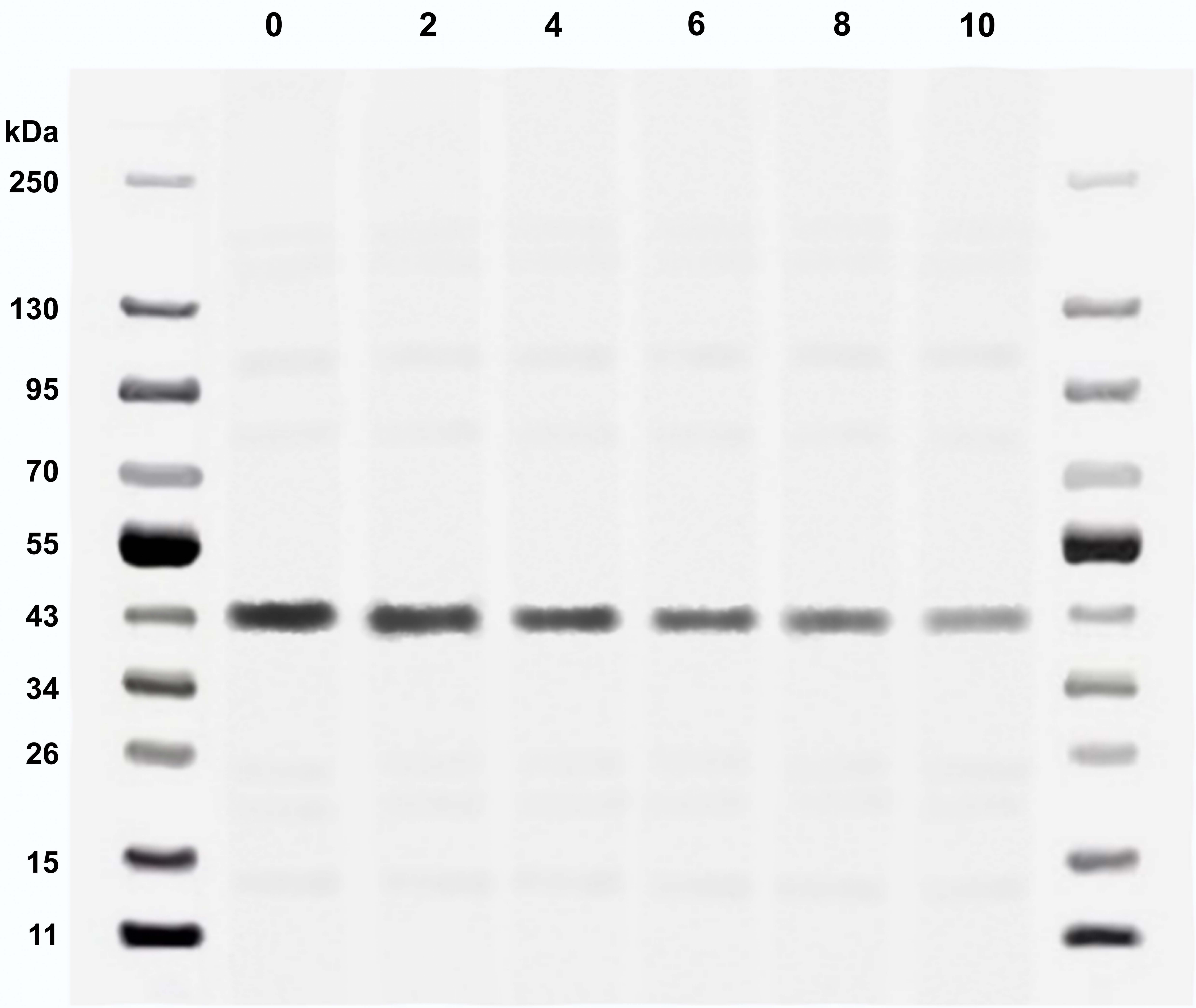

Fig 7B Dose dependence CHI3L1

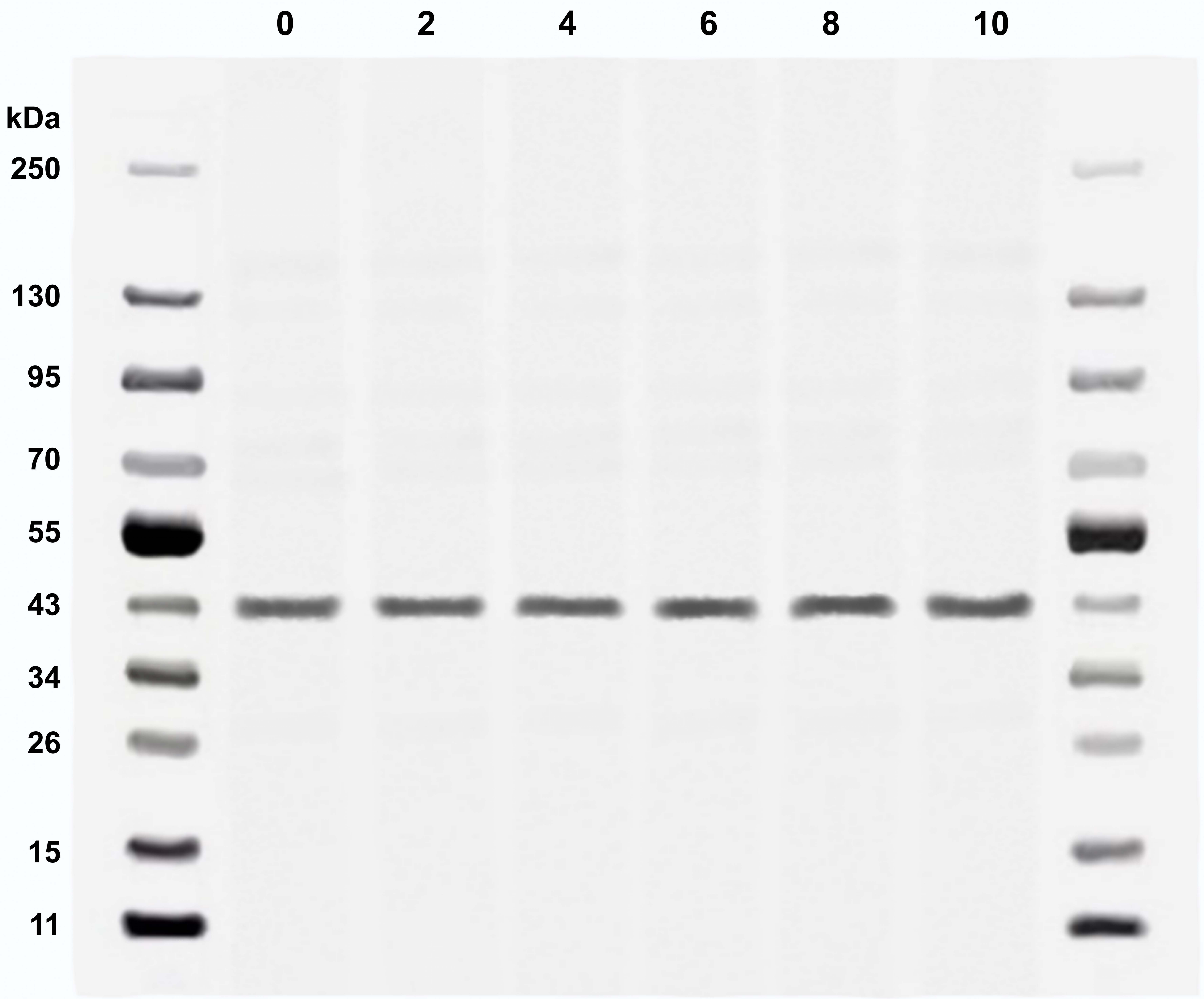

Fig 7B Dose dependence GAPDH

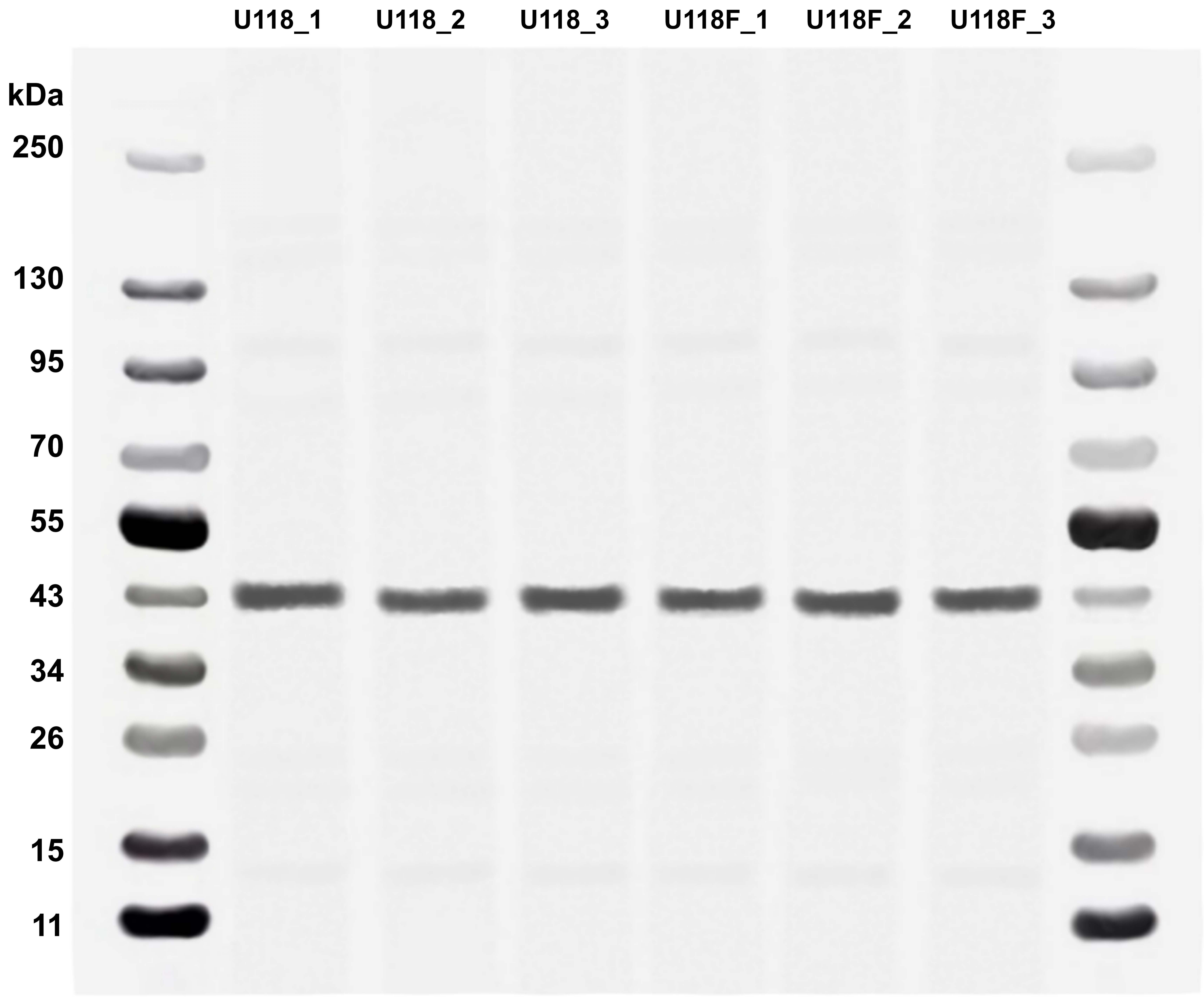

Fig 7C CHI3L1

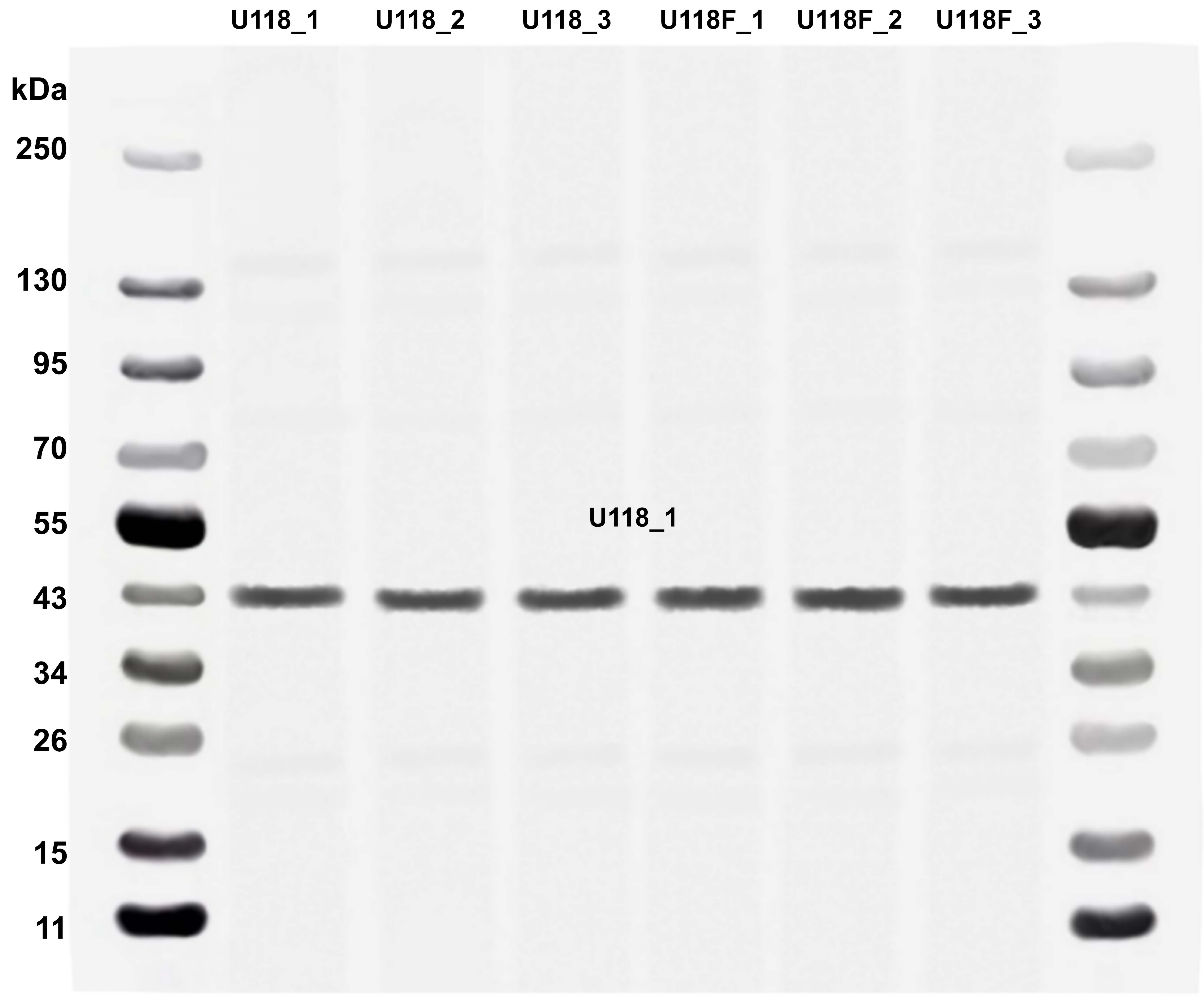
U118_1
U118_2
U118_3
U118F_1
U118F_2
U118F_3
kDa
250
130
95
70
55
43
34
26
15
11
U118_1

Fig 7C GAPDH

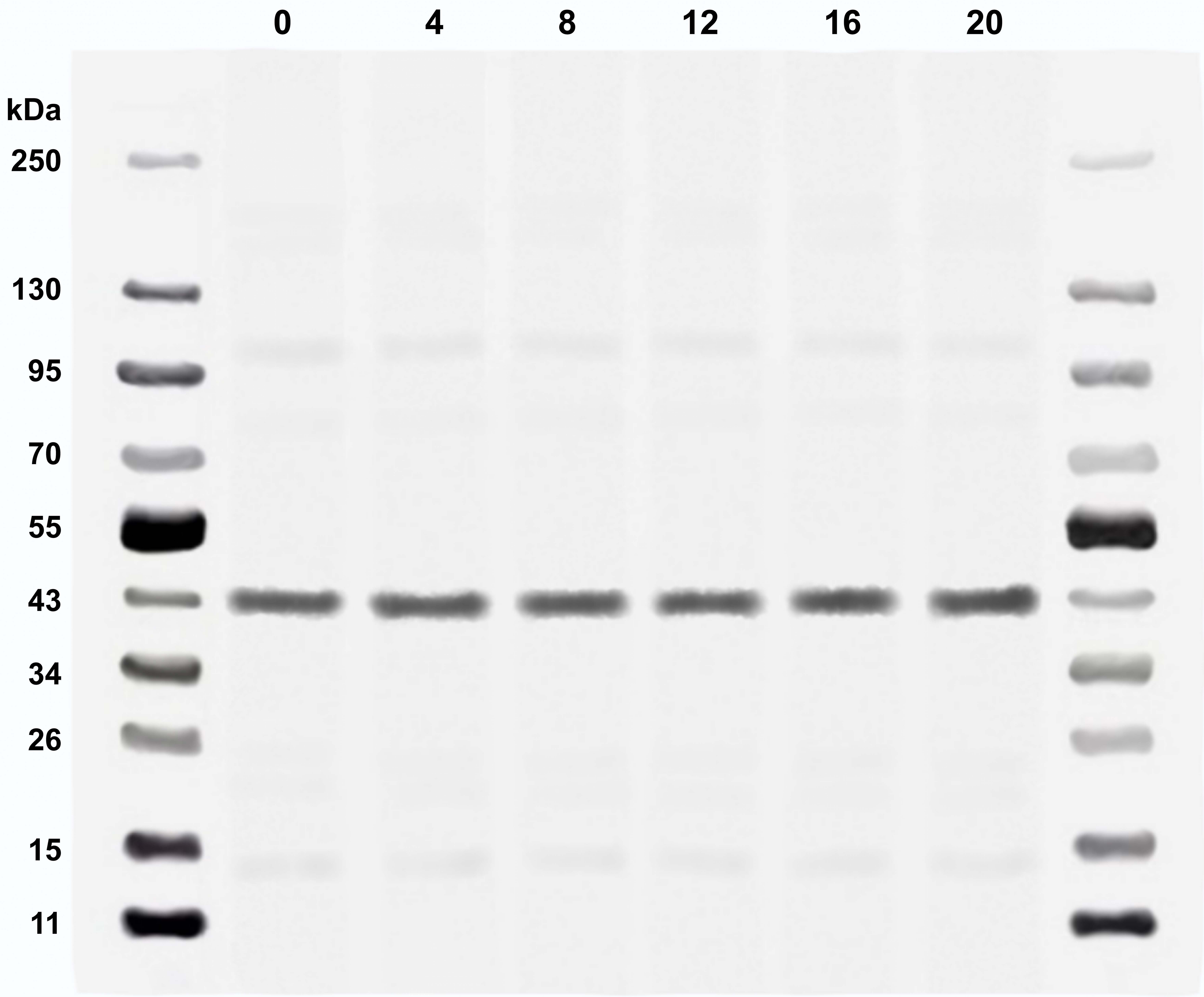

Fig 7D Does dependence CHI3L1

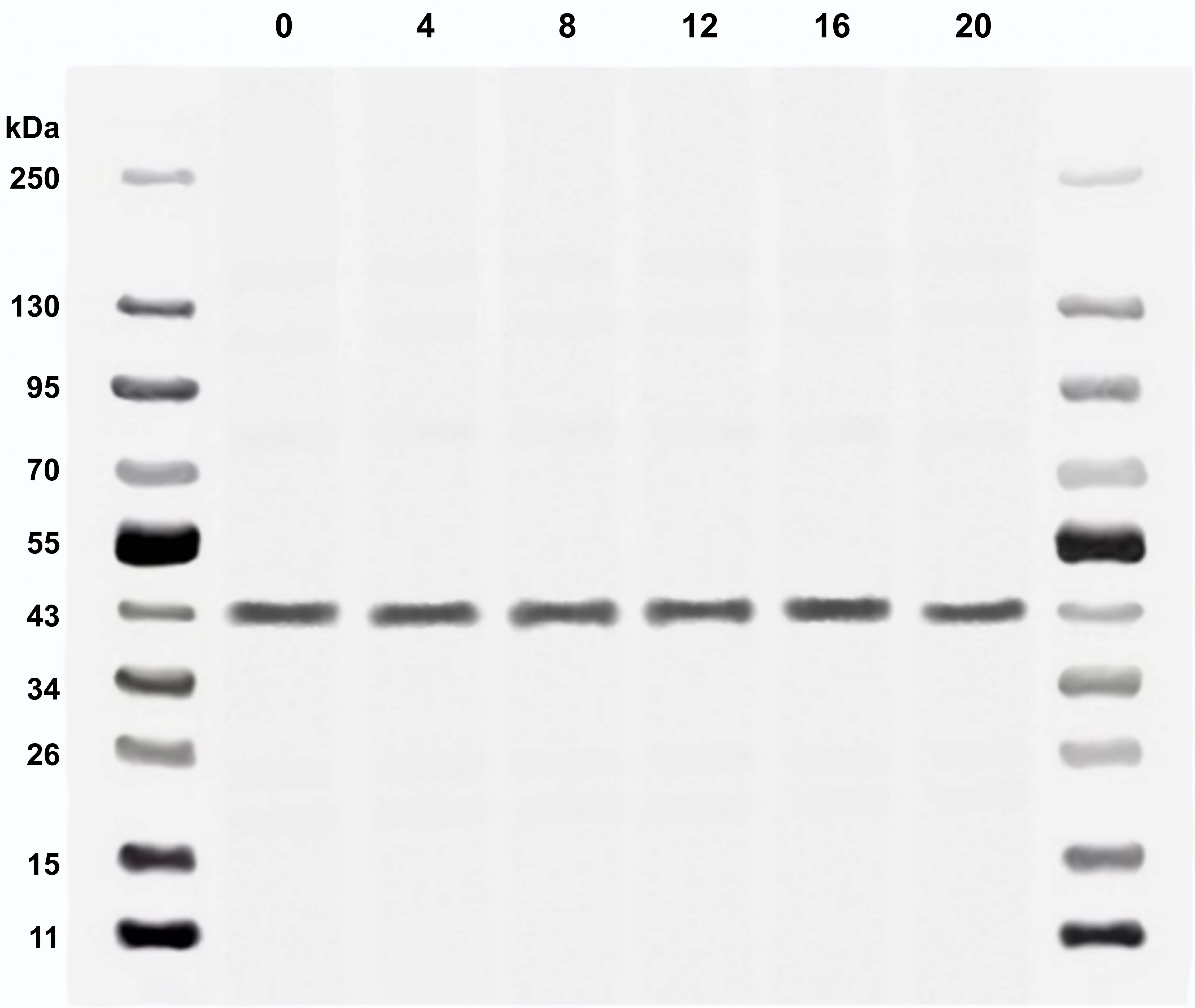

Fig 7D Dose dependence GAPDH

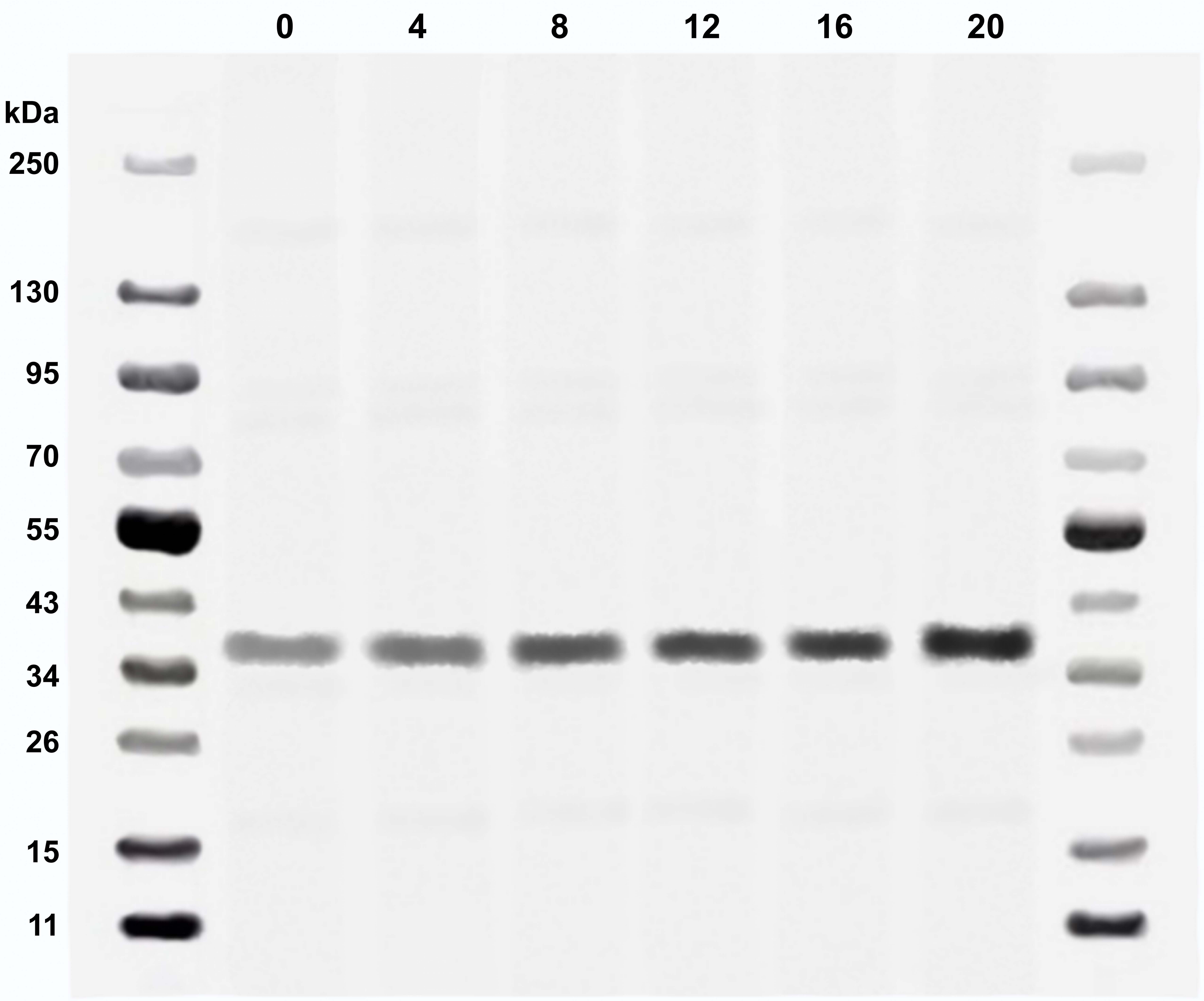

S4A Fig JUNB

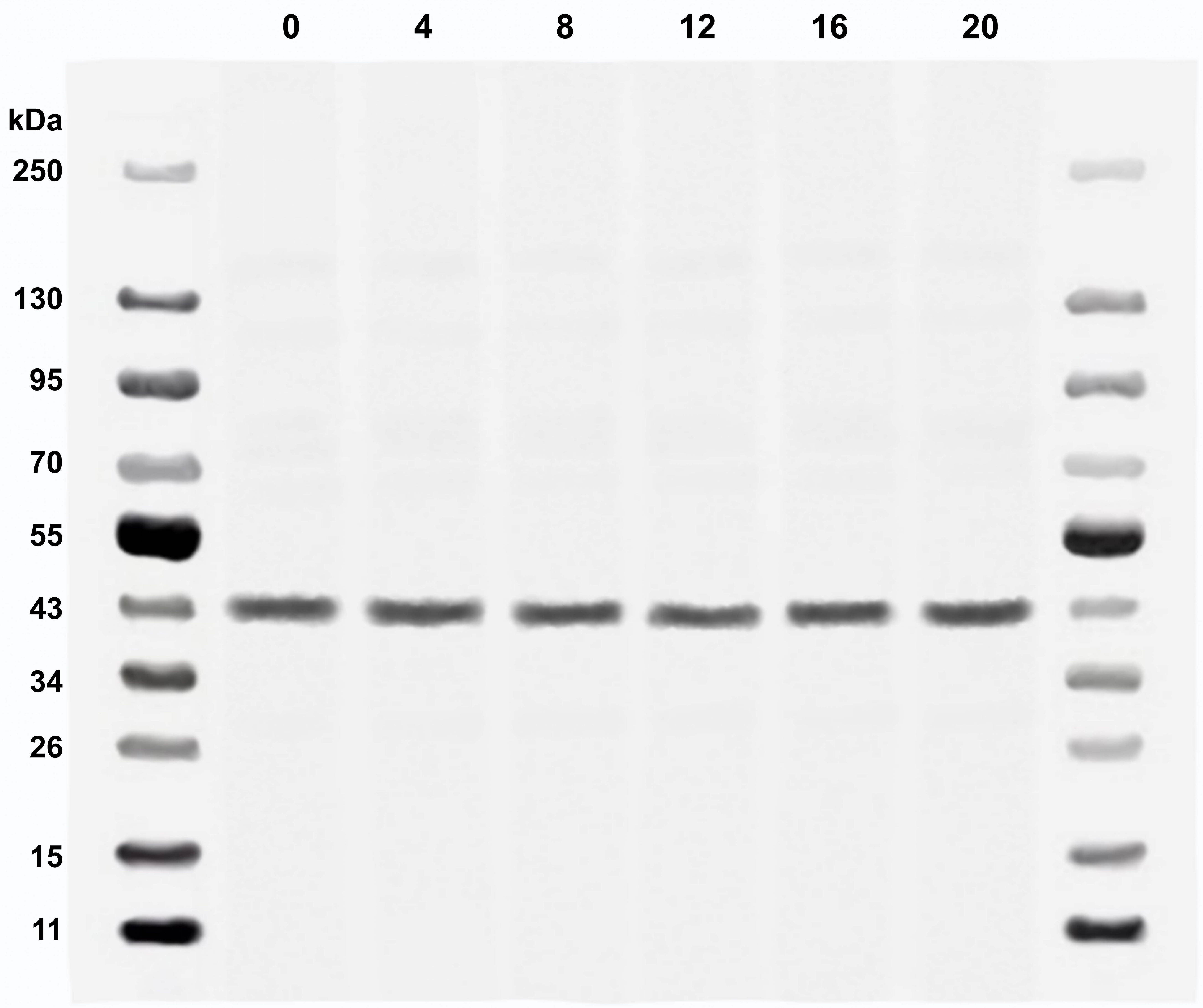

S4A Fig GAPDH

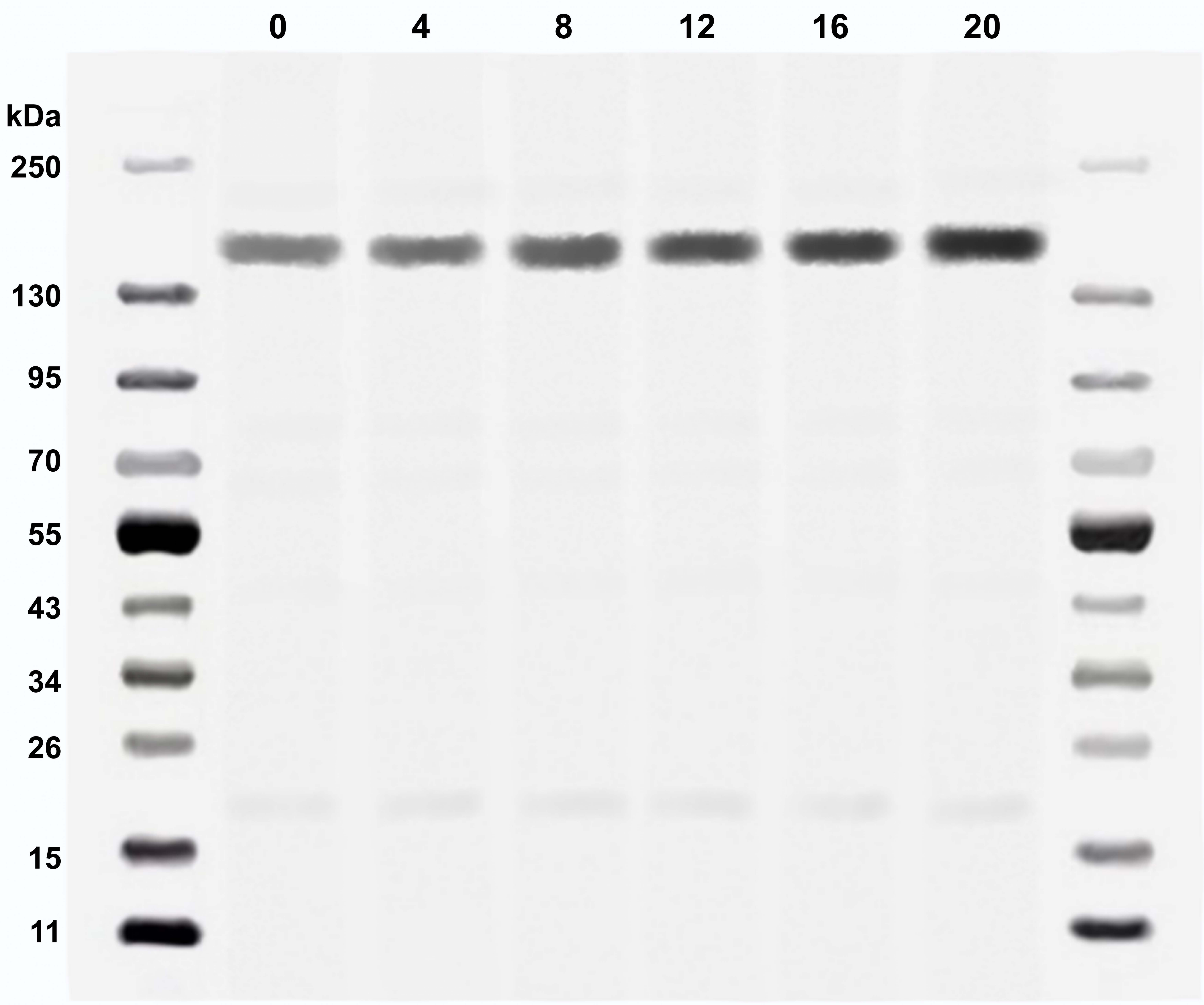

S4B Fig

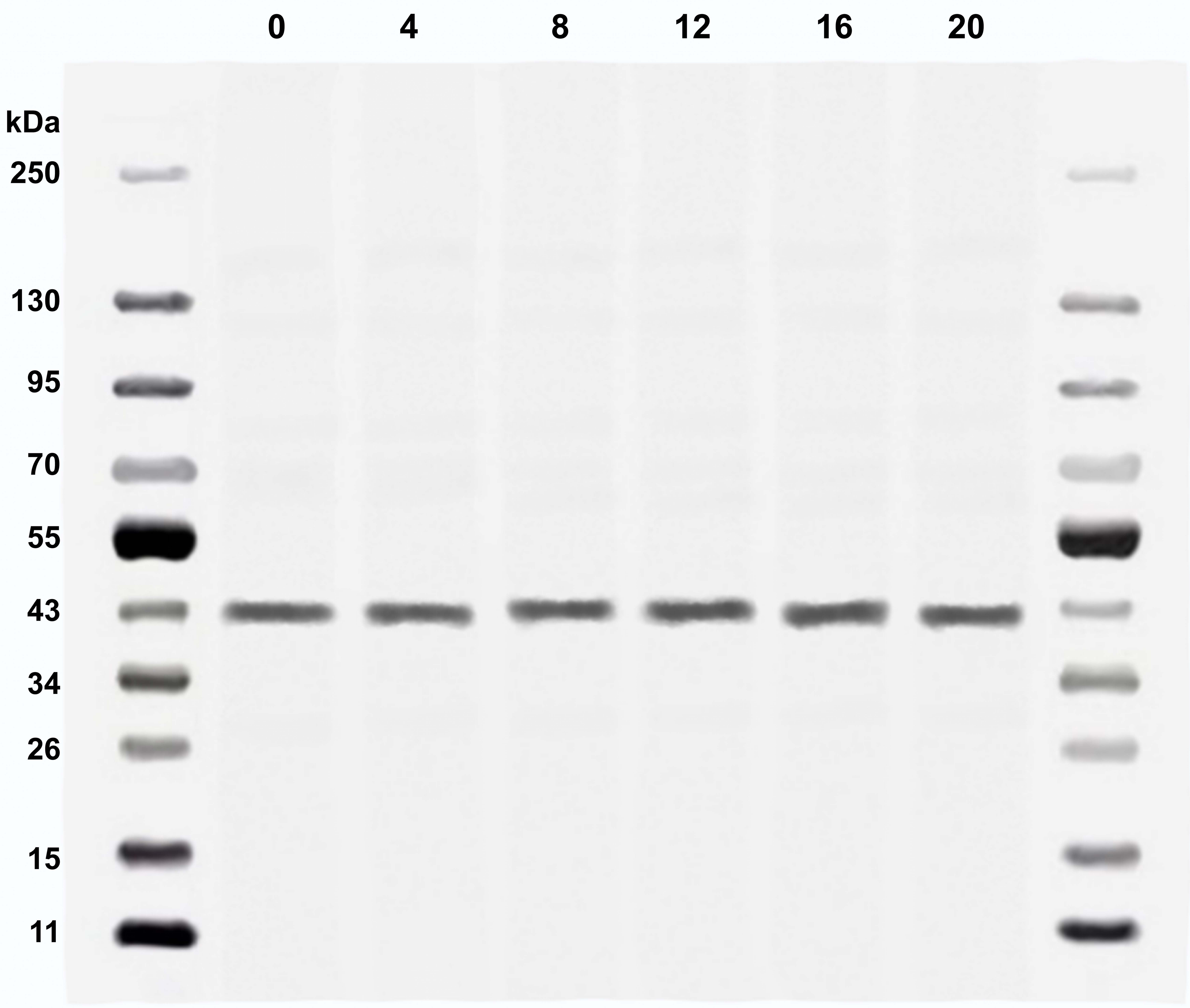

S4B Fig GAPDH
