# Supplementary material for: Host-defence caerin 1.1 and 1.9 peptides suppress glioblastoma U87 and U118 cell proliferation through the modulation of mitochondrial respiration and induce the downregulation of CHI3L1
Source: PLoS One. 2024 Jun 7;19(6):e0304149. doi: 10.1371/journal.pone.0304149 (PMC11161062; doi:10.1371/journal.pone.0304149)
Supplement: Attachment — Submitted filename: Response to reviewers.docx [file pone.0304149.s011.docx]

Dear editor and reviewers,

Thank you very much for editing and reviewing our manuscript. Please find our point-by-point answers to the questions raised by the reviewers. Changes to the manuscript are shown in red font in the "'Revised Manuscript with Track Changes".

**Editor comments:**

1. It would be better to insert scales into Fig. 4 so the reader could get idea on the scales of the system under study.

**RESPONSE:** Scales have been inserted in revised Fig 4.

**Reviewer #1**

1. It would be better to insert scales into Fig. 4 so the reader could get idea on the scales of the system under study.

**RESPONSE:** We have added scale bar of the system under study to revised Fig 4 and one sentence in the figure legend.

The responses provided above address the comments from the editor and the reviewers. We would like to express our gratitude for your valuable input and time in reviewing our work.

Sincerely

Tianfang Wang, Ph.D.
